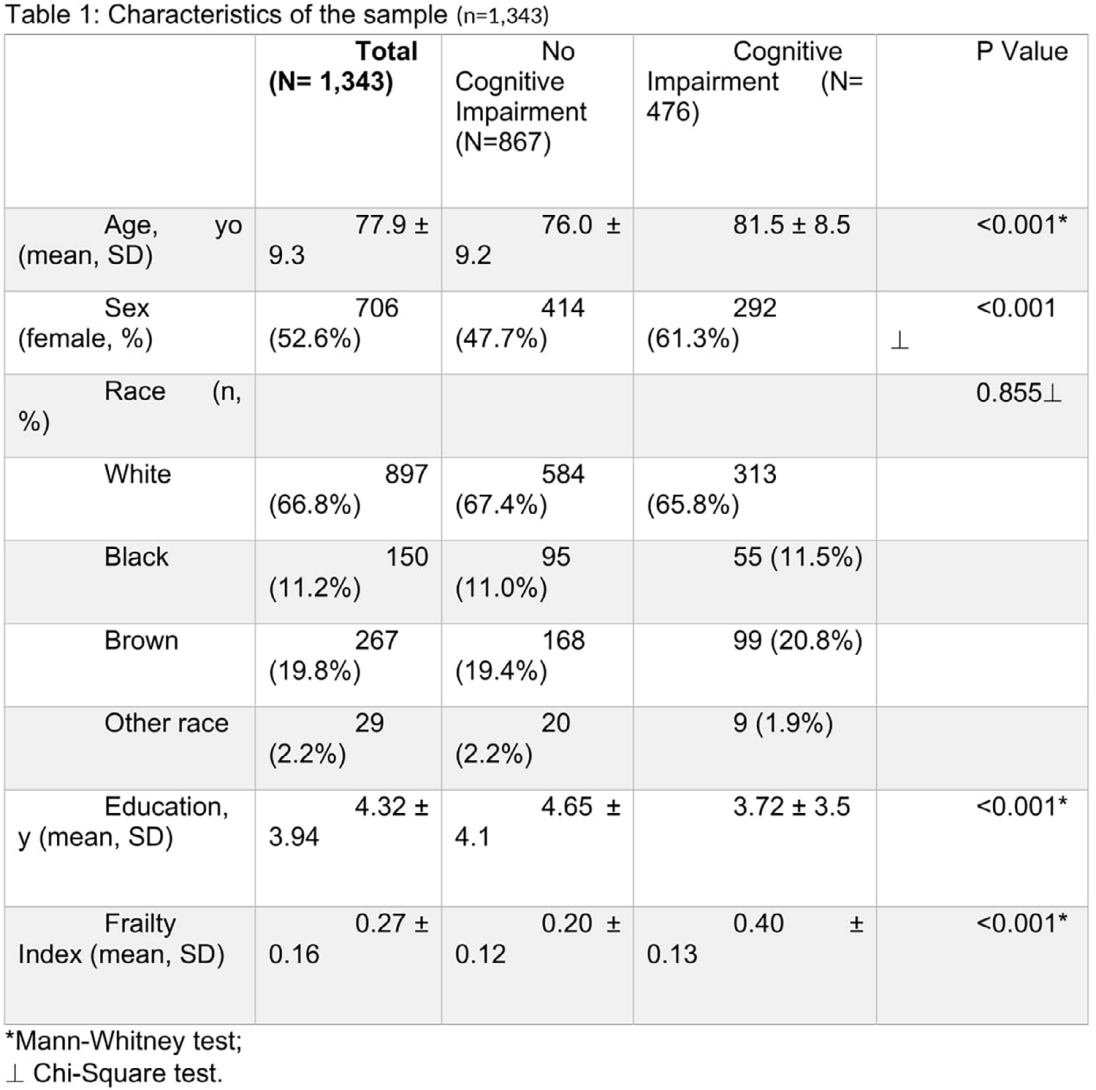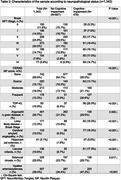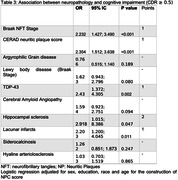# Association of frailty and neuropathology of neurodegenerative and brain vascular diseases

**DOI:** 10.1002/alz.091773

**Published:** 2025-01-09

**Authors:** Felipe Bozi Soares, Renata Elaine Paraizo Leite, Lea T. Grinberg, Vitor Ribeiro Paes, Roberta Diehl Rodriguez, Carlos Augusto Pasqualucci, Wilson Jacob‐Filho, Claudia Kimie Suemoto

**Affiliations:** ^1^ Physiopathology in Aging Laboratory (LIM‐22), Department of Internal Medicine, University of Sao Paulo Medical School, Brazil, São Paulo, São Paulo Brazil; ^2^ Physiopathology in Aging Laboratory (LIM‐22), University of São Paulo Medical School, São Paulo, São Paulo Brazil; ^3^ Memory & Aging Center, Department of Neurology, University of California in San Francisco, San Francisco, CA USA; ^4^ Physiopathology in Aging Laboratory (LIM‐22), Department of Internal Medicine, University of Sao Paulo Medical School, São Paulo, São Paulo Brazil; ^5^ Cognitive and Behavioral Neurology Unit ‐ University of São Paulo, São Paulo Brazil; ^6^ University of São Paulo Medical School, São Paulo Brazil; ^7^ Division of Geriatrics, University of São Paulo Medical School, São Paulo Brazil; ^8^ Division of Geriatrics, Department of Internal Medicine, University of Sao Paulo Medical School, São Paulo, São Paulo Brazil; ^9^ Division of Geriatrics, University of São Paulo Medical School, São Paulo, São Paulo Brazil; ^10^ Biobank for Aging Studies of the University of São Paulo, São Paulo Brazil

## Abstract

**Background:**

Frailty, characterized by increased physical vulnerability, is associated with a higher incidence and severity of cognitive impairment and also a higher burden of neurodegenerative and cerebrovascular diseases. This study investigates the association between frailty and neurodegenerative and cerebrovascular pathologies.

**Method:**

Cross‐sectional analysis using clinical and neuropathological data from individuals aged 60 or older, enrolled in the Biobank for Aging Studies between 2004 and 2023. A 42‐item frailty index was constructed. Cognitive impairment was defined as a clinical dementia rating score (CDR) of 0.5 or over and participants were stratified according to cognitive status. Linear regression models, adjusting for age, sex, education and race, explored the association between frailty and neuropathology, including Alzheimer´s disease (AD), argyrophilic grain disease (AGD), Lewy‐type pathology (LBP), hippocampal sclerosis, cerebral amyloid angiopathy (CAA), lacunar infarcts, hyaline arteriosclerosis, TDP‐43 pathology and a neuropathological comorbidity score (NPC).

**Results:**

We examined data from 1.343 subjects. The group with cognitive impairment was older, predominantly female, had lower education, a higher frailty index, and no race differences (Table 1). This group also exhibited a higher prevalence of all neuropathologies previously described (Table 2). In adjusted analyses, frailty was associated with AD Braak staging (β = 0.022, 95% CI=0.017; 0.028, p<0.001 ), CERAD score (β = 0.021, CI 95% = 0.013; 0.029 p<0.001), CAA (β = 0.065, 95% CI = 0.039; 0.092 p<0.001), LBD (β = 0.051, 95% CI = 0.027; 0.075 p<0.001), hippocampal sclerosis (β= 0.053, CI 95% = 0.008; 0.100 p=0.022), lacunar infarcts (β=0.071, CI 95%= 0.046; 0.097 p<0.001), siderocalcinosis (β=0.033, CI 95%= 0.015; 0.051 p<0.001), hyaline arteriosclerosis (β=0.065, CI 95%= 0.050‐0.081 p<0.001) and NPC score (Table 3) (β=0.032, CI 95%= 0.024; 0.040 p<0.001). Frailty was not associated with AGD and TDP‐43.

**Conclusion:**

Frailty was associated with several neuropathological markers of neurodegenerative and cerebrovascular diseases. More studies are warranted to investigate how this association relates to relevant outcomes such as cognitive impairment.